# *CCDC69* Regulates Adipocyte Differentiation and Cellular Morphology in SGBS Cells—A Human Preadipocyte Cell Line

**DOI:** 10.3390/cells15191782

**Published:** 2026-09-30

**Authors:** Takeo Iwata

**Affiliations:** Department of Functional Morphology, Faculty of Pharmaceutical Sciences, Niigata University of Pharmacy and Medical and Life Sciences, 265-1 Higashijima, Akiha-ku, Niigata City 956-8603, Japan; iwata@nupals.ac.jp; Tel.: +81-250255269

**Keywords:** CCDC69, SGBS cells, adipogenesis

## Abstract

*CCDC69* was previously identified as a gene upregulated during adipogenic differentiation of human Simpson–Golabi–Behmel syndrome (SGBS) preadipocytes but not mouse 3T3-L1 preadipocytes. Here, the role of *CCDC69* in adipogenesis was investigated using SGBS cells. *CCDC69* expression during adipogenic differentiation was assessed by quantitative reverse transcription PCR and Western blotting. *CCDC69* function was examined by adenovirus-mediated overexpression and knockdown, followed by analyses of lipid accumulation, adipocyte marker expression, cell morphology, and cytoskeletal organization. *CCDC69* expression increased during adipogenesis. *CCDC69* overexpression enhanced adipogenesis, as indicated by increased lipid accumulation, a higher proportion of cells containing lipid droplets, and increased expression of adipocyte marker genes and proteins. Unexpectedly, *CCDC69* knockdown also enhanced adipogenesis. Time-course analysis showed that *CCDC69* overexpression suppressed *PPARG* mRNA expression during early adipogenesis but increased its expression at later stages, whereas knockdown increased *PPARG* expression at later stages. *CCDC69* overexpression induced a flattened cell morphology accompanied by reduced fibrillar α-tubulin structures and F-actin stress fibers, whereas knockdown increased the number of small, round cells. These findings suggest that the effects of *CCDC69* on adipogenesis may vary across the differentiation process in SGBS cells and are associated with adipocyte morphology and cytoskeletal organization.

## 1. Introduction

Obesity is a condition characterized by excessive accumulation of adipose tissue and is often associated with type 2 diabetes, dyslipidemia, and hypertension. Obesity and its associated complications contribute to the development of atherosclerosis and increase the risk of cerebrovascular and cardiovascular diseases [1]. The prevalence of obesity is increasing worldwide, and the prevention and treatment of obesity are urgent public health issues.

Excessive accumulation of adipose tissue is thought to result from hypertrophy of individual adipocytes and increased adipogenesis [2]; therefore, elucidating the molecular mechanisms underlying these processes may lead to the identification of molecular targets for anti-obesity drugs. Although extensive research has been conducted on these mechanisms, much of it has relied on animal models or non-human cell lines such as 3T3-L1 cells. However, adipogenic differentiation is not completely conserved between humans and mice, and several factors have been reported to exhibit human-specific expression or differential effects during adipogenesis [3,4,5]. For example, LMO3 is selectively upregulated during human adipocyte differentiation and promotes this process [6]. Furthermore, while D-dopachrome tautomerase, an adipokine, inhibits adipogenesis in human preadipocytes, it has no effect on adipogenesis in 3T3-L1 cells [7]. Thus, research using human cells is important for identifying molecular targets for anti-obesity drugs in humans.

In a previous study, a comprehensive microarray analysis was conducted to identify genes whose expression is induced during the adipogenic differentiation of SGBS cells and 3T3-L1 cells [5]. By comparing the results, genes whose expression is induced in SGBS cells but not in 3T3-L1 cells were identified. One of these genes, *CCDC69*, was selected for further investigation in this study.

CCDC69 is a coiled-coil domain-containing protein involved in microtubule regulation and has been proposed to function as a scaffold protein involved in protein–protein interactions [8]. CCDC69 is anchored to the plasma membrane through myristoylation of its N-terminal glycine residue [9]. CCDC69 has been implicated in cytokinesis, central spindle assembly, and the regulation of microtubule dynamics during mitosis, partly through interactions with KIF2C/MCAK and the chromosome passenger complex [8,9]. Dysregulation of CCDC69 causes severe mitotic abnormalities, including microtubule disorganization, delayed chromosome alignment, and chromosome scattering [8,9]. In addition, *CCDC69* has also been reported as a prognostic marker and regulator of chemotherapy resistance in several types of cancer, including breast cancer, ovarian cancer, and sarcoma [10,11,12,13,14,15,16]. Publicly available transcriptomic data from the Human Protein Atlas indicate that CCDC69 is expressed in human adipose tissue, including adipocytes and B cells [17,18]. Genome-wide expression analysis has also shown that CCDC69 expression is modulated during adipogenic differentiation of human adipose-derived stromal cells [19]. However, the role of *CCDC69* in adipocyte function and adipogenesis has not yet been elucidated.

In this study, the role of *CCDC69* in the adipogenic differentiation of SGBS cells was investigated. By manipulating *CCDC69* expression through overexpression and knockdown, its effects on adipocyte differentiation and cell morphology were evaluated. Furthermore, changes in the organization of microtubules and F-actin during adipogenesis were examined.

## 2. Materials and Methods

### 2.1. Cell Line

SGBS cells (RRID: CVCL_GS28), a human preadipocyte cell line established from the subcutaneous adipose tissue of an infant with Simpson-Golabi-Behmel syndrome [20], were kindly provided by Dr. Martin Wabitsch (Ulm University Medical Center, Ulm, Germany) in September 2012. The cells were routinely tested and confirmed to be free of Mycoplasma contamination by DAPI staining and PCR. SGBS cells were selected as the experimental model because they are a well-established human preadipocyte cell line with a reproducible capacity for adipogenic differentiation and are particularly suitable for investigating human-specific mechanisms of adipogenesis [20,21].

### 2.2. Ethics Statement

This study used only the established SGBS cell line and did not involve human participants, collection of human tissues, or identifiable human data. Therefore, institutional ethical approval and informed consent were not applicable to this study.

### 2.3. Construction of Adenoviruses

Adenoviruses expressing CCDC69 fused to a FLAG tag at the C-terminus and two shRNAs targeting *CCDC69* mRNA (shCCDC69) were constructed as previously described [5,22]. Briefly, the coding region of *CCDC69* lacking the stop codon was amplified from SGBS adipocyte cDNA by PCR. A DNA fragment containing the FLAG cDNA sequence was then added to the 3′ end of the *CCDC69* coding sequence, and the resulting construct was inserted into the pAxCAwtit cosmid vector (Takara, Shiga, Japan). Recombinant adenoviral genomic DNA was excised from the cosmid and transfected into HEK293 cells to produce adenoviruses. An adenovirus generated from the intact pAxCAwtit vector was used as the control adenovirus (vehicle).

Cosmids for adenovirus production based on the pAV[shRNA]-EGFP vector and expressing shCCDC69 #1 (target sequence: 5′-GACTGGTCAAAGGACTCTTAT-3′; vector ID: VB900140-4270zbc) or shCCDC69 #2 (target sequence: 5′-CACGCTGGCCATTTGACATAA-3′; vector ID: VB900212-2306snw), as well as a negative-control shRNA (shNC; target sequence: 5′-CCTAAGGTTAAGTCGCCCTCG-3′; vector ID: VB010000-9479yzr), were purchased from VectorBuilder (Chicago, IL, USA). The corresponding adenoviruses were produced in the same manner.

### 2.4. Cell Culture and Adipogenic Induction

SGBS cells at passages 35–38 were used in the experiments. Cells were cultured in 6-well plates using a 1:1 mixture of Dulbecco’s Modified Eagle Medium (DMEM) and Ham’s F12 (Sigma-Aldrich, St Louis, MI, USA), supplemented with 10% fetal bovine serum (FBS; Corning, Corning, NY, USA), 33 μM biotin, and 17 μM pantothenic acid (both from Sigma-Aldrich). Cells were grown to confluence and infected with adenovirus at a multiplicity of infection (MOI) of 10. Adipogenic differentiation was induced 24 h post-infection. Based on microscopic observation of GFP fluorescence, approximately 100% of cells exhibited GFP fluorescence following transduction with the shRNA adenovirus under the experimental conditions used. The transduction efficiency of the CCDC69-FLAG adenovirus was not independently quantified.

For adipogenic induction, cells were washed twice with PBS and switched to a serum-free 1:1 mixture of DMEM and Ham’s F12 containing 20 nM insulin, 100 nM cortisol, 0.2 nM triiodothyronine, 10 μg/mL transferrin, 25 nM dexamethasone, 250 μM 3-isobutyl-1-methylxanthine, and 2 μM troglitazone. Cells were cultured in this induction medium for 4 days at 37 °C in a humidified atmosphere with 5% CO_2_. Subsequently, they were maintained in serum-free DMEM/Ham’s F12 (1:1) medium supplemented with 20 nM insulin, 100 nM cortisol, 0.2 nM triiodothyronine, and 10 μg/mL transferrin. The maintenance medium was replaced every 3 days, and cells were cultured until the indicated time points.

### 2.5. Evaluation of Adipogenesis

Adipocyte differentiation was evaluated based on the expression of adipocyte markers, lipid accumulation, and the percentage of cells containing lipid droplets. SGBS cells were infected with each adenovirus for 24 h and then subjected to adipogenic induction. At the indicated time points after adipogenic induction, total RNA and protein were extracted from the cells, and the expression levels of adipocyte markers were determined by quantitative reverse transcription PCR (qRT-PCR) and Western blotting, respectively.

At 4 or 9 days after adipogenic induction, SGBS cells were fixed with 4% paraformaldehyde in phosphate-buffered saline (PBS) and stained with Oil Red O to evaluate lipid accumulation or with 4,6-diamidino-2-phenylindole dihydrochloride (DAPI) and Sudan III to determine the percentage of cells containing lipid droplets. For quantitative analysis of lipid accumulation, Oil Red O was eluted with isopropanol, and the absorbance was measured at 500 nm using a spectrophotometer (Ultrospec 6300 Pro; GE Healthcare, Chicago, IL, USA). To determine the percentage of cells containing lipid droplets, the stained cells were observed under a fluorescence microscope (Nikon Eclipse Ts2; Nikon, Tokyo, Japan), and images were captured using a ToupCAM camera (ToupTek Photonics, Hangzhou, China). For each independent experiment, images were acquired from at least three randomly selected fields of view at ×100 magnification. The percentage of cells containing lipid droplets was calculated by dividing the total number of Sudan III-positive cells by the total number of DAPI-positive cells across the analyzed fields. For cell number analysis, DAPI-positive cells were counted in the same manner on days 0, 1, 4, and 9 after adipogenic induction. The percentages or cell number obtained from the individual fields were averaged within each independent experiment, and the resulting mean value was used as one biological replicate for statistical analysis.

### 2.6. qRT-PCR

Total RNA was extracted from the cells, and cDNA was synthesized using the ReverTra Ace qPCR RT Kit (Toyobo, Osaka, Japan) according to the manufacturer’s instructions. qRT-PCR was performed using a Thermal Cycler Dice Real Time System (Takara, Shiga, Japan) or a StepOne Real-Time PCR System (Thermo Fisher Scientific, Foster City, CA, USA) with Thunderbird SYBR qPCR Mix (Toyobo) and gene-specific primer sets. The amplification program consisted of an initial denaturation at 95 °C for 15 s, followed by 40 cycles of denaturation at 95 °C for 15 s and annealing/extension at 60 °C for 1 min. The specificity of each primer set was confirmed by dissociation curve analysis after amplification. The nucleotide sequences of the primer sets are listed in Supplementary Table S1. The mRNA expression levels of the respective genes were normalized to that of *GAPDH*.

### 2.7. Western Blotting

Cell lysates were prepared using a lysis buffer containing 50 mM Tris-HCl (pH 7.4), 150 mM NaCl, 1 mM ethylenediaminetetraacetic acid (EDTA), 1% Triton X-100, and Complete Mini protease inhibitor cocktail (Roche, Basel, Switzerland). The lysates were separated by sodium dodecyl sulfate-polyacrylamide gel electrophoresis (SDS-PAGE) and subsequently transferred onto polyvinylidene difluoride (PVDF) membranes (Immobilon Transfer Membranes; Millipore, Bedford, MA, USA). The membranes were stained with Ponceau S staining solution (Beacle, Kyoto, Japan), when applicable, photographed, and destained before blocking with Blocking One (Nacalai Tesque, Kyoto, Japan). The membranes were then incubated overnight at 4 °C with the following primary antibodies: mouse anti-FLAG M2 antibody (1:1000; Sigma-Aldrich), rabbit anti-CCDC69 antibody (1:2000; Thermo Fisher Scientific), mouse anti-PPARγ antibody (1:5000; Proteintech, Rosemont, IL, USA), or mouse anti-adiponectin antibody (1:1000; Proteintech). The membranes were then incubated with horseradish peroxidase-conjugated anti-rabbit or anti-mouse IgG secondary antibodies (Jackson ImmunoResearch, West Grove, PA, USA) for 40 min at room temperature. Immunoreactive signals were visualized using Immobilon Western Detection Reagent (Millipore) and a Luminograph III imaging system (Atto, Tokyo, Japan). Band intensities were quantified using ImageJ software 1.53t (National Institutes of Health, Bethesda, MD, USA), and total protein levels visualized by Ponceau S staining were used for normalization. Uncropped Western blot images with the positions of representative molecular weight markers indicated are provided in Supplementary Figure S1.

### 2.8. Immunofluorescent Staining

The cells cultured on glass coverslips placed in 6-well plates were fixed in 4% formaldehyde in PBS at 37 °C for 10 min. Cells used for α-tubulin staining were fixed in PEM buffer (0.1 M PIPES [pH 7.0], 1 mM ethylene glycol-bis(β-aminoethyl ether)-N,N,N′,N′-tetraacetic acid, 1 mM MgSO_4_). Fixed cells were permeabilized with PBS containing 0.1% Triton X-100 for 10 min and then incubated with Blocking One (Nacalai Tesque) for 1 h. The cells were incubated overnight at 4 °C with mouse anti-FLAG antibody (1:500), rabbit anti-CCDC69 antibody (1:200), or rabbit anti-α-tubulin antibody (1:200; Medical & Biological Laboratories, Tokyo, Japan). The cells were then incubated for 40 min at 37 °C with Alexa Fluor 488 goat anti-mouse IgG (Abcam, Cambridge, UK), Alexa Fluor 568 goat anti-rabbit IgG (Abcam), Alexa Fluor 488 goat anti-rabbit IgG (Thermo Fisher Scientific), or Alexa Fluor 594 goat anti-mouse IgG (Thermo Fisher Scientific), together with 1 µg/mL DAPI, 1 µM BODIPY 493/503 (Selleck, Houston, TX, USA) or Phalloidin-iFluo 594 Conjugate (1:1000; AAT Bioquest, Pleasanton, CA, USA), as appropriate. The cells were observed using a confocal fluorescence microscope (LSM700; Zeiss, Oberkochen, Germany).

Cell morphology was evaluated using ImageJ software 1.53t. For all image-based analyses, including cell area, circularity, F-actin, and α-tubulin morphology, fluorescence images from at least three randomly selected fields at ×100 magnification were acquired for each independent experiment. Measurements from multiple fields were averaged within each independent experiment, and the resulting value was used as one biological replicate for statistical analysis.

Cell boundaries were manually traced, and cell area and circularity were measured. Circularity was calculated using the standard ImageJ formula, 4π × area/perimeter^2^, where a value of 1.0 represents a perfect circle.

In the CCDC69-F overexpression experiment, cells with a mean FLAG grayscale value of 5,000 or higher were classified as high FLAG-expressing cells (FLAG-high), while cells with values below that threshold were classified as FLAG-low/negative cells. This threshold was selected based on the fluorescence intensity at which FLAG-positive cells could be clearly identified during blinded assessment. For F-actin analysis, cells containing three or more clearly distinguishable stress fibers within the cell were classified as stress fiber-positive, whereas the remaining cells were classified as stress fiber-negative. For α-tubulin analysis, cells were classified as having normal or abnormal morphology. Cells showing relatively weak tubulin staining in the nuclear region with fibers radiating from the perinuclear region throughout the cytoplasm were classified as having normal tubulin morphology; all other cells were classified as abnormal. Cells in which the morphology could not be clearly evaluated, such as cells overlapping with other cells, were also classified as abnormal. The classification of F-actin and α-tubulin morphology was performed by an independent observer who was blinded to the experimental groups.

### 2.9. Statistical Analysis

Each experiment was independently performed at least three times using independently prepared cell cultures, each of which was separately induced to differentiate, and representative data are shown where appropriate. Data are presented as the mean ± standard error (SE). Measurements from multiple wells or fields at each time point were averaged within each independent experimental run, and the resulting mean value was used as one biological replicate for statistical analysis. Statistical analyses were performed using Student’s *t*-test for comparisons between two groups and one-way ANOVA followed by Dunnett’s multiple-comparisons test for comparisons of three or more groups with a control group, and two-way ANOVA followed by Tukey’s HSD test for analyses involving two factors. Differences were considered statistically significant at *p* < 0.05. Exact *p* values, effect sizes, 95% confidence intervals, and the number of biological replicates are provided in Supplementary Tables S2 and S3.

## 3. Results

### 3.1. CCDC69 Expression Is Induced During Adipogenesis in SGBS Cells

**Figure 1:**
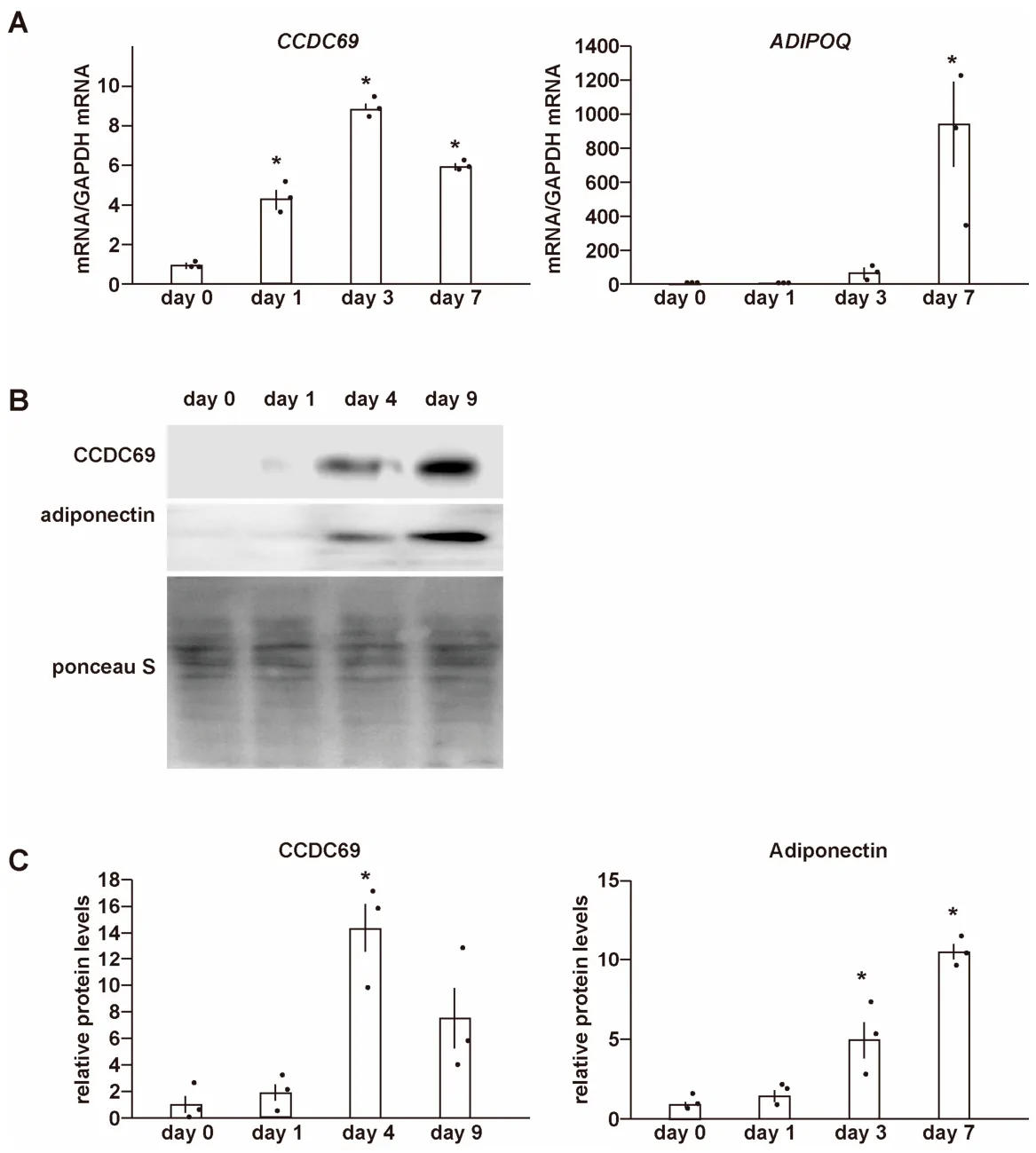
*CCDC69* expression during adipogenesis in SGBS cells. (**A**) Relative *CCDC69* and *ADIPOQ* mRNA expression in SGBS cells on days 0, 1, 3, and 7 after adipogenic induction, normalized to the level on day 0. (**B**) Representative Western blotting of CCDC69 and adiponectin expression in SGBS cell lysates on days 0, 1, 4, and 9 after adipogenic induction. Ponceau S staining was used as a loading control. (**C**) Densitometric quantification of CCDC69 and adiponectin expression normalized to total protein levels visualized by Ponceau S staining and expressed relative to the level on day 0 (set to 1). Individual data points are shown in the graphs. Asterisks indicate *p* < 0.05. Exact *p* values, effect sizes, 95% confidence intervals, and the number of biological replicates are provided in Supplementary Table S2.

First, to verify the microarray analysis results, the expression pattern of *CCDC69* mRNA during the adipogenic differentiation of SGBS cells was examined using qRT-PCR. *CCDC69* mRNA expression began to increase on day 1 after adipogenic induction and peaked on day 3 (Figure 1A). CCDC69 protein expression showed a similar pattern (Figure 1B,C). Adiponectin mRNA and protein levels increased during adipogenic differentiation, confirming the progression of adipogenesis (Figure 1A–C). These findings suggest that CCDC69 may play a role in the adipogenic differentiation of SGBS cells.

### 3.2. CCDC69 Overexpression Enhances Adipogenesis in SGBS Cells

**Figure 2:**
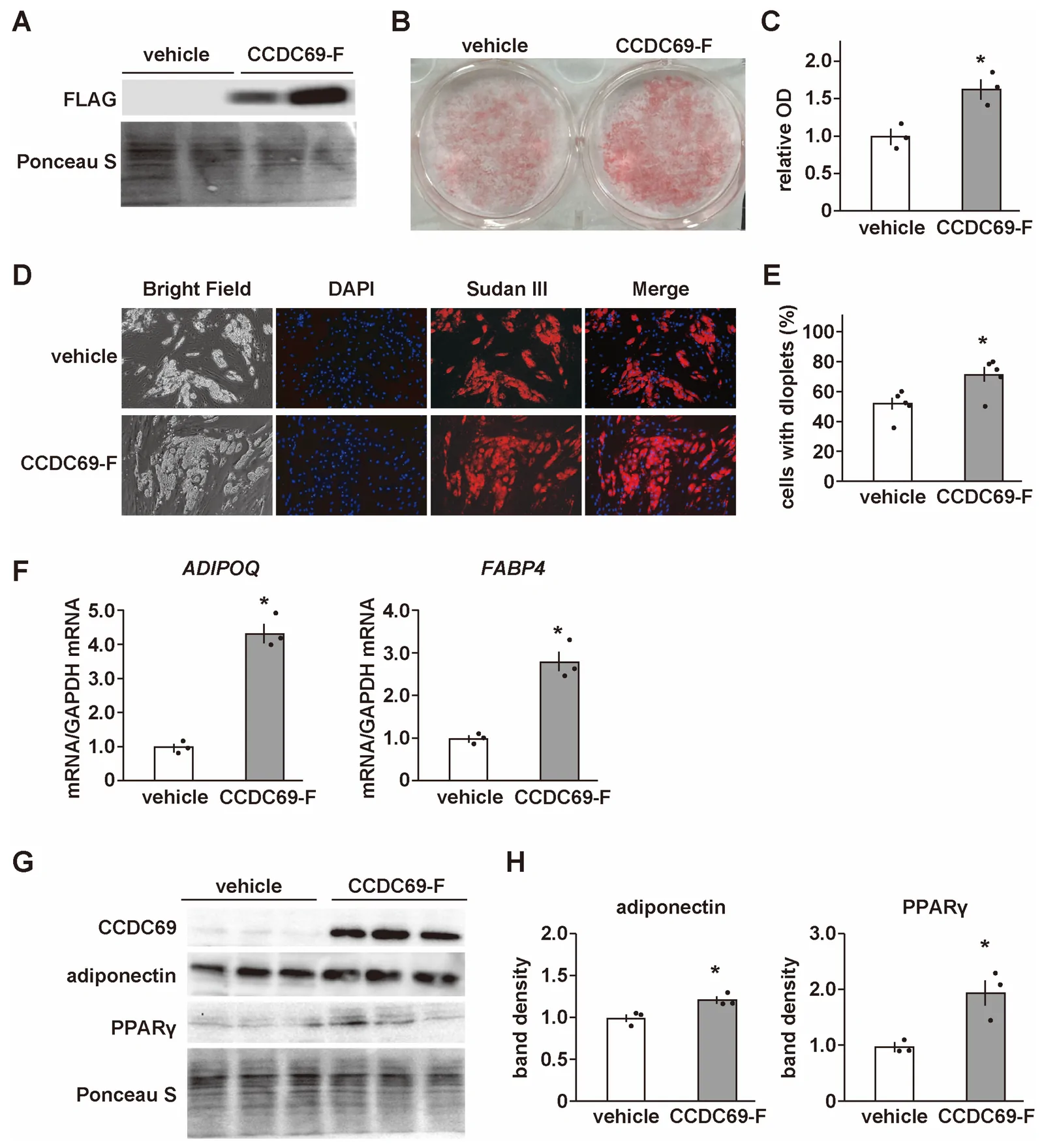
Effects of *CCDC69* overexpression on adipogenesis in SGBS cells. SGBS cells were infected with an adenovirus expressing the CCDC69-FLAG fusion protein (CCDC69-F) or the control adenovirus (vehicle) for 24 h, followed by adipogenic induction. Adipogenesis was evaluated on day 9 after adipogenic induction. (**A**) Expression of the CCDC69-FLAG fusion protein in SGBS cells after adipogenic induction. Cell lysates were analyzed by Western blotting using an anti-FLAG antibody. Ponceau S staining was used as a loading control. (**B**) Representative images of SGBS cells stained with Oil Red O. (**C**) Quantification of lipid accumulation based on the absorbance of eluted Oil Red O. Values are expressed relative to those in vehicle-treated cells. (**D**) Representative images (×100 magnification) of SGBS cells stained with DAPI and Sudan III. (**E**) Percentage of cells containing lipid droplets in SGBS cells subjected to adipogenic induction. (**F**) Relative mRNA expression of the adipocyte marker genes *ADIPOQ* and *FABP4* in SGBS cells subjected to adipogenic induction. (**G**) Representative Western blotting of CCDC69, adiponectin, and PPARγ in lysates from SGBS cells subjected to adipogenic induction. Ponceau S staining was used as a loading control. (**H**) Densitometric quantification of each protein, normalized to total protein levels visualized by Ponceau S staining and expressed relative to the level in vehicle-treated cells (set to 1). Individual data points are shown in the graphs. Asterisks indicate *p* < 0.05. Exact *p* values, effect sizes, 95% confidence intervals, and the number of biological replicates are provided in Supplementary Table S2.

The effects of *CCDC69* overexpression on adipogenesis in SGBS cells were investigated. SGBS cells were infected with an adenovirus expressing the CCDC69-FLAG fusion protein or vehicle for 24 h, followed by adipogenic induction. In this overexpression system, no significant difference in cell number was observed between the control group and the overexpression group up to day 9 after the induction of adipogenesis (Supplementary Figure S2A), suggesting that CCDC69 overexpression did not affect cell number in this experiment. Expression of the CCDC69-FLAG fusion protein was confirmed in SGBS cells on day 9 after adipogenic induction (Figure 2A). SGBS cells expressing the CCDC69-FLAG fusion protein showed greater Oil Red O staining than control cells (Figure 2B,C). Furthermore, the percentage of cells containing lipid droplets was evaluated by DAPI and Sudan III staining. *CCDC69* overexpression significantly increased the proportion of cells containing lipid droplets (Figure 2D,E). In addition, *CCDC69* overexpression significantly increased the mRNA expression levels of the adipocyte marker genes *ADIPOQ* and *FABP4* (Figure 2F) and increased the protein levels of adiponectin and peroxisome proliferator-activated receptor γ (PPARγ) (Figure 2G,H). These results indicate that *CCDC69* overexpression enhances adipogenesis in SGBS cells.

### 3.3. CCDC69 Knockdown Also Enhances Adipogenesis in SGBS Cells

**Figure 3:**
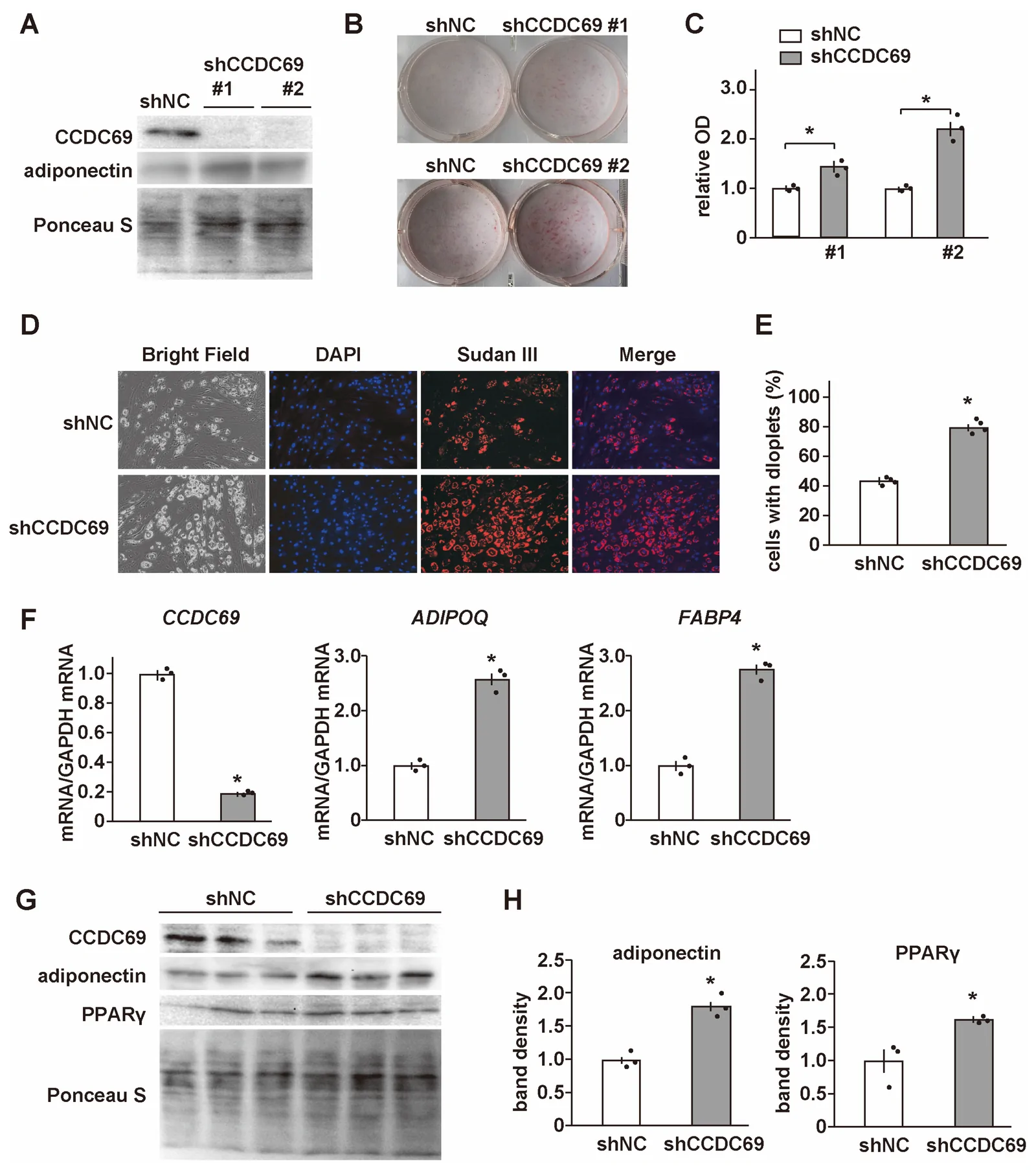
Effects of *CCDC69* knockdown on adipogenesis in SGBS cells. SGBS cells were infected with adenoviruses expressing two different shRNAs targeting *CCDC69* mRNA (shCCDC69 #1 and #2) or a negative-control shRNA (shNC) for 24 h, followed by adipogenic induction. Adipogenesis was evaluated on day 9 after adipogenic induction. shCCDC69 #2 was used for the experiments shown in panels D–H. (**A**) Expression of CCDC69 and adiponectin in SGBS cells after adipogenic induction. Cell lysates were analyzed by Western blotting using an anti-CCDC69 or anti-adiponectin antibody. Ponceau S staining was used as a loading control. (**B**) Representative images of SGBS cells stained with Oil Red O. (**C**) Quantification of lipid accumulation based on the absorbance of eluted Oil Red O. Values are expressed relative to those in shNC-treated cells. (**D**) Representative images (×100 magnification) of SGBS cells stained with DAPI and Sudan III. (**E**) Percentage of cells containing lipid droplets in SGBS cells subjected to adipogenic induction. (**F**) Relative mRNA expression of *CCDC69* and the adipocyte marker genes *ADIPOQ* and *FABP4* in SGBS cells subjected to adipogenic induction. Values are expressed relative to those in shNC-treated cells. (**G**) Representative Western blotting of CCDC69, adiponectin, and PPARγ in lysates from SGBS cells subjected to adipogenic induction. Ponceau S staining was used as a loading control. (**H**) Densitometric quantification of each protein, normalized to total protein levels visualized by Ponceau S staining and expressed relative to the level in shNC-treated cells (set to 1). Individual data points are shown in the graphs. Asterisks indicate *p* < 0.05. Exact *p* values, effect sizes, 95% confidence intervals, and the number of biological replicates are provided in Supplementary Table S2.

Next, the effects of *CCDC69* knockdown on adipogenesis in SGBS cells were examined. SGBS cells were infected with adenoviruses expressing shRNAs targeting different sequences of *CCDC69* mRNA (shCCDC69 #1 and shCCDC69 #2) or a negative-control shRNA (shNC) for 24 h, followed by adipogenic induction. No significant difference in cell number was observed between the control group and the knockdown groups up to day 9 after the induction of adipogenesis (Supplementary Figure S2B). On day 9 after adipogenic induction, both shCCDC69 constructs were confirmed to suppress CCDC69 expression (Figure 3A) and increased the intensity of Oil Red O staining (Figure 3B,C). shCCDC69 #2, which produced a greater increase in Oil Red O staining, was chosen for the subsequent experiments. shCCDC69 #2 significantly increased the proportion of cells containing lipid droplets (Figure 3D,E). Furthermore, *CCDC69* knockdown reduced *CCDC69* mRNA expression and increased the mRNA expression levels of *ADIPOQ* and *FABP4* (Figure 3F) and elevated the protein levels of adiponectin and PPARγ (Figure 3G,H). These results suggest that *CCDC69* knockdown, like *CCDC69* overexpression, promotes adipogenesis in SGBS cells. Notably, shCCDC69 #1 exhibited a similar phenotype (Supplementary Figure S3).

### 3.4. CCDC69 Overexpression Alters PPARG Expression at Different Stages of Adipogenesis

**Figure 4:**
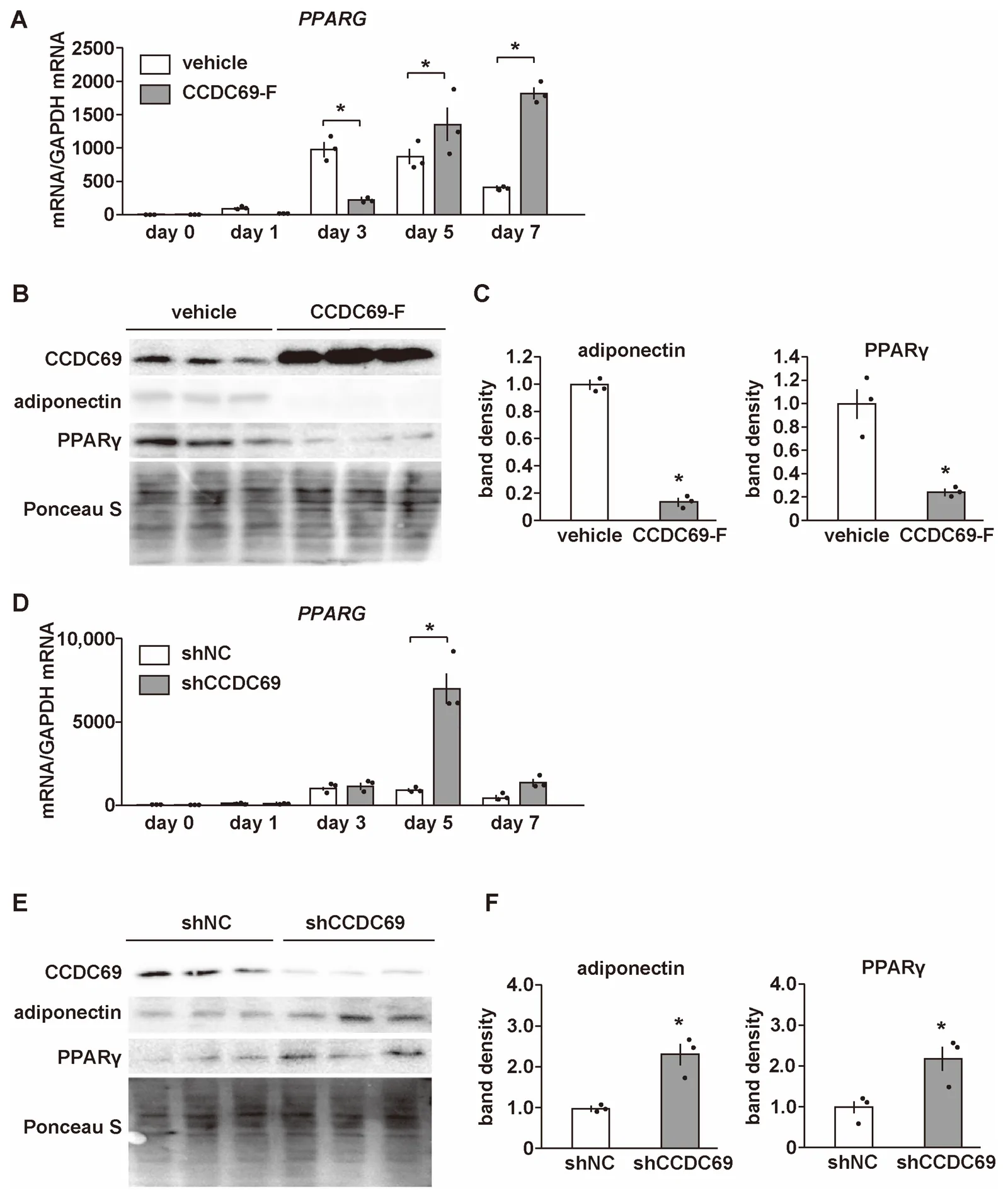
Effects of *CCDC69* overexpression or knockdown on PPARγ expression during SGBS adipogenesis. SGBS cells were infected with an adenovirus expressing the CCDC69-FLAG fusion protein (CCDC69-F; gray columns in (**A**,**C**)), shCCDC69 #2 (shCCDC69; gray columns in (**D**,**F**)), or the corresponding control adenovirus (vehicle or shNC; white columns) for 24 h, followed by adipogenic induction. (**A**,**D**) *PPARG* mRNA levels on days 0, 1, 3, 5, and 7 post-induction, relative to control on day 0 (set to 1). Data were analyzed by two-way ANOVA with treatment, time, and their interaction as fixed effects, followed by Tukey’s HSD test. (**B**,**E**) Representative Western blots of CCDC69, adiponectin, and PPARγ on day 4 post-induction; Ponceau S staining was used as a loading control. (**C**,**F**) Densitometric quantification normalized to total protein levels visualized by Ponceau S staining, relative to respective control (set to 1). Individual data points are shown in the graphs. Asterisks indicate *p* < 0.05. Exact *p* values, effect sizes, 95% confidence intervals, and the number of biological replicates are provided in Supplementary Table S2.

To elucidate the mechanism by which *CCDC69* overexpression and knockdown exert similar effects on adipogenesis in SGBS cells, their effects on the expression pattern of PPARγ during adipogenesis were examined. *PPARG* mRNA expression increased from day 1 after adipogenic induction in vehicle-treated cells. In *CCDC69*-overexpressing cells, however, this induction was significantly suppressed on day 1 (approximately 0.13-fold) and remained suppressed on day 3 (approximately 0.24-fold), when *PPARG* mRNA expression reached its peak in vehicle-treated cells. Interestingly, from day 5, *CCDC69* overexpression conversely resulted in a significant increase in *PPARG* mRNA expression (Figure 4A). Consistent with the inhibitory effects observed at the mRNA level on days 1 and 3, *CCDC69* overexpression also reduced PPARγ protein levels on day 4 after adipogenic induction (Figure 4B,C). Furthermore, adiponectin protein levels were also reduced, suggesting that *CCDC69* overexpression suppresses adipogenesis during the early stages of adipogenic differentiation.

In contrast, suppression of *CCDC69* expression tended to increase *PPARG* mRNA expression from day 3 after adipogenic induction, with a significant increase observed on day 5 (Figure 4D). It also increased PPARγ and adiponectin protein levels on day 4 after adipogenic induction (Figure 4E,F). Together with the results shown in Figure 3, these findings suggest that *CCDC69* knockdown promotes adipogenesis.

### 3.5. SGBS Cells Strongly Expressing CCDC69 Exhibit a Flattened Morphology

CCDC69 is a microtubule-associated protein that binds to microtubules and has been reported to be involved in the regulation of microtubule dynamics [8,9]. Furthermore, during adipogenesis, the cytoskeleton undergoes reorganization, leading to changes in cell shape and size [23,24]. Therefore, whether CCDC69 affects cell morphology during adipogenesis in SGBS cells was examined.

**Figure 5:**
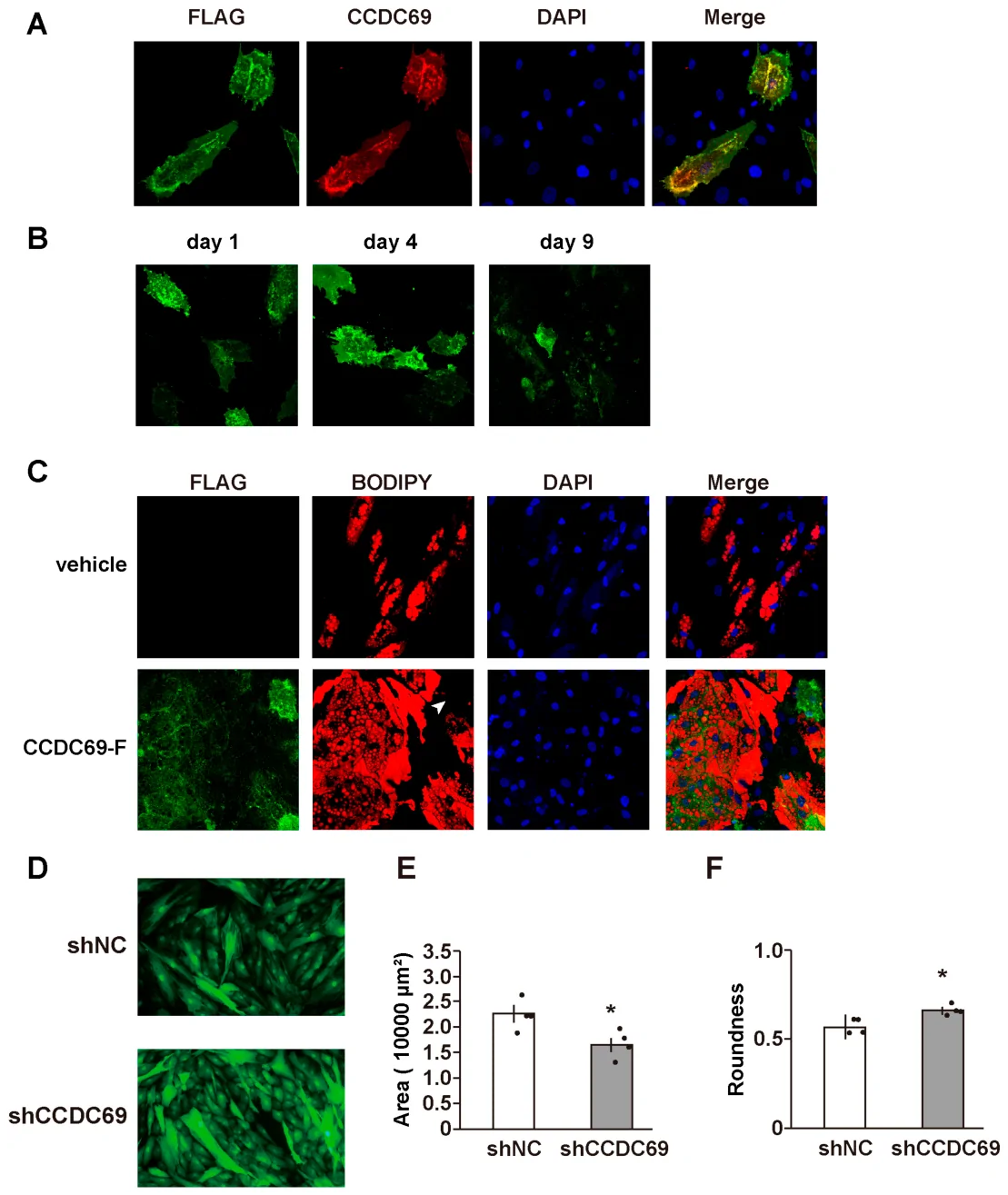
Effects of *CCDC69* overexpression or knockdown on cell shape during adipogenesis in SGBS cells. SGBS cells were infected with an adenovirus expressing the CCDC69-FLAG fusion protein (CCDC69-F), shCCDC69 #2 (shCCDC69), or the corresponding control adenovirus (vehicle or shNC) for 24 h, followed by adipogenic induction. (**A**) Representative images (×200 magnification) of SGBS cells overexpressing CCDC69-F on day 1 after adipogenic induction, stained with anti-FLAG or anti-CCDC69 antibodies. (**B**) Representative images (×100 magnification) of SGBS cells overexpressing CCDC69-F on days 1, 4, and 9 after adipogenic induction, stained with an anti-FLAG antibody. (**C**) Representative images of SGBS cells overexpressing CCDC69-F on day 9 after adipogenic induction, stained with an anti-FLAG antibody, DAPI, and BODIPY. (**D**) Representative images (×100 magnification) of SGBS cells expressing shCCDC69 or shNC on day 4 after adipogenic induction. (**E**) Quantification of cell area in SGBS cells expressing shCCDC69 or shNC on day 4 after adipogenic induction. (**F**) Quantification of cell circularity in SGBS cells expressing shCCDC69 or shNC on day 4 after adipogenic induction. Individual data points are shown in the graphs. Asterisks indicate *p* < 0.05. Exact *p* values, effect sizes, 95% confidence intervals, and the number of biological replicates are provided in Supplementary Table S2.

SGBS cells were infected with an adenovirus expressing CCDC69-FLAG for 24 h, followed by adipogenic induction. First, on day 1 after adipogenic induction, the FLAG-positive signal was confirmed to coincide with the localization of CCDC69 detected by the anti-CCDC69 antibody (Figure 5A). Next, the morphology of CCDC69-FLAG-expressing cells during adipogenesis was examined. Cells exhibiting particularly strong FLAG signals showed a flattened morphology from day 1 through day 9 after adipogenic induction (Figure 5B). On day 9 after adipogenic induction, FLAG-positive signals were observed in cells containing lipid droplets, with the signals localized outside the lipid droplets. However, some cells exhibiting strong FLAG signals remained flat and did not contain lipid droplets, and lipid droplets were not observed in areas showing strong FLAG signals (Figure 5C).

Furthermore, the effects of *CCDC69* knockdown on cell morphology were examined. Although no obvious morphological differences were observed between shCCDC69- and shNC-expressing cells on day 4 after adipogenic induction (Figure 5D), quantitative analysis revealed that shCCDC69-expressing cells had a smaller cell area and higher circularity than shNC-expressing cells (Figure 5E,F).

### 3.6. Overexpression of CCDC69 Alters Cytoskeletal Organization

**Figure 6:**
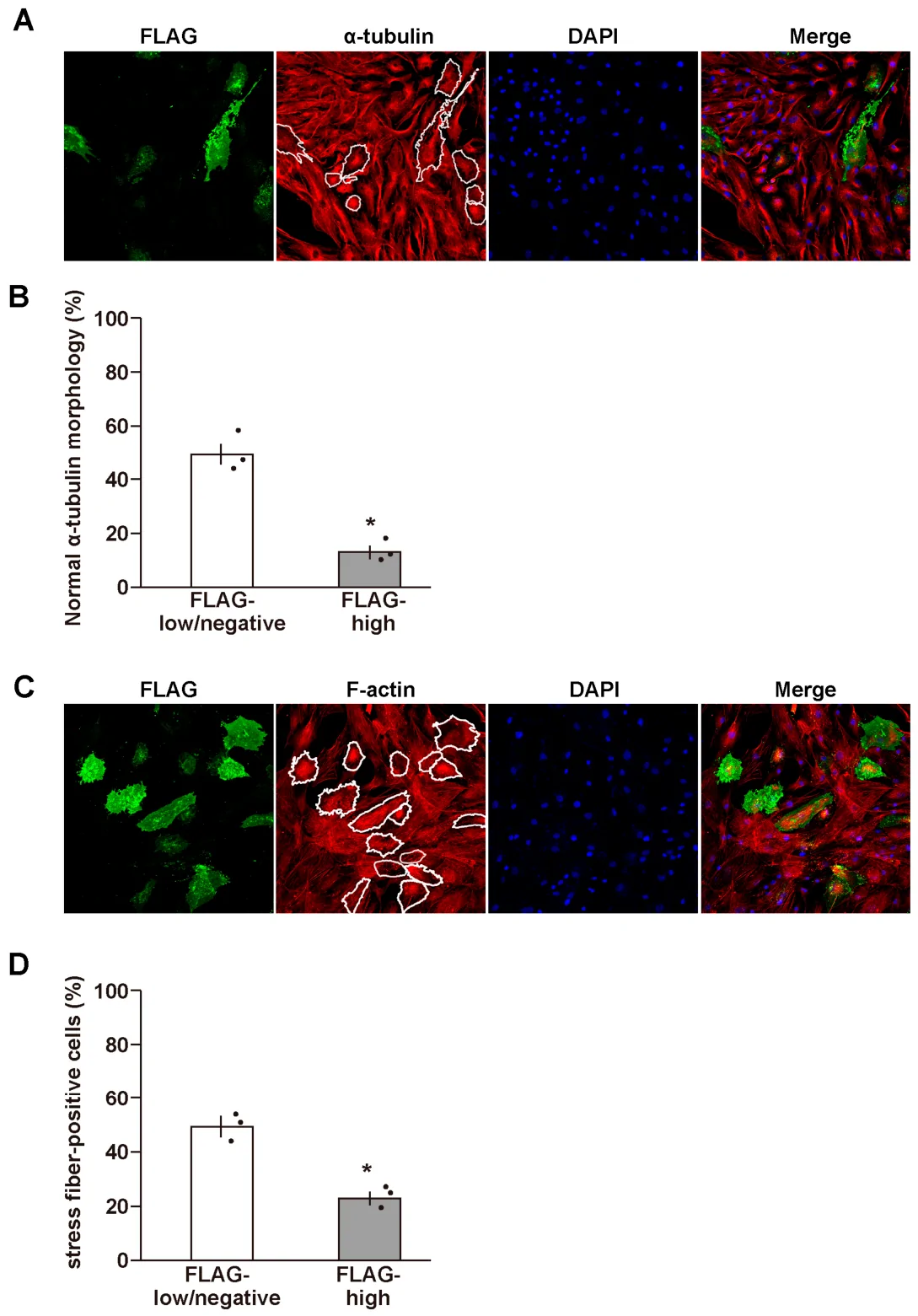
Effects of *CCDC69* overexpression on the cytoskeleton in SGBS cells. SGBS cells were infected with an adenovirus expressing the CCDC69-FLAG fusion protein (CCDC69-F) or vehicle for 24 h, followed by adipogenic induction. (**A**) Representative images (×100 magnification) of SGBS cells overexpressing CCDC69-F on day 1 after adipogenic induction, stained with anti-FLAG and anti-α-tubulin antibodies. White outlines indicate FLAG-high cells used for analysis. (**B**) Quantification of normal α-tubulin morphology (%) in FLAG-low/negative and FLAG-high cells. (**C**) Representative images (×100 magnification) of SGBS cells overexpressing CCDC69-F on day 1 after adipogenic induction, stained with an anti-FLAG antibody and phalloidin. White outlines indicate FLAG-high cells used for analysis. (**D**) Quantification of stress fiber-positive cells (%) in FLAG-low/negative and FLAG-high cells. Individual data points are shown in the graphs. Asterisks indicate *p* < 0.05. Exact *p* values, effect sizes, 95% confidence intervals, and the number of biological replicates are provided in Supplementary Table S2.

Finally, the cytoskeletal organization of cells overexpressing *CCDC69* was examined on day 1 after adipogenic induction. In SGBS cells exhibiting strong FLAG signals, the fibrillar structure of α-tubulin was reduced or absent (Figure 6A,B). FLAG-high cells accounted for 10.2 ± 0.8% (mean ± SE, *n* = 3) of the analyzed cells in the α-tubulin analysis. Furthermore, phalloidin staining revealed that F-actin stress fibers were also reduced or absent (Figure 6C,D). FLAG-high cells accounted for 11.5 ± 2.5% (mean ± SE, *n* = 3) of the analyzed cells in the F-actin analysis. The localization of these F-actin structures coincided with the FLAG-positive signal (Figure 6C).

## 4. Discussion

In the present SGBS adipogenesis model, the mRNA expression levels of adipocyte marker genes, including *ADIPOQ* and *FABP4*, peaked on day 7 post-induction, whereas those of *PPARG* and *CEBPA* peaked on day 3. In a previous study, microarray analysis of SGBS cell adipogenesis identified *CCDC69* as a gene whose expression was markedly upregulated following adipogenic induction, with increases of 20.12-, 18.82-, and 10.81-fold on days 1, 3, and 7, respectively, compared with baseline levels prior to induction (day 0) [5]. In the present study, qRT-PCR analysis confirmed that *CCDC69* expression exhibited a temporal pattern similar to that of *PPARG*, peaking during the early phase of adipogenesis. Consistent with these findings, genome-wide analysis has shown that *CCDC69* expression is upregulated on days 1 and 7 during adipogenesis in human adipose-derived stromal cells and returns to basal levels on days 14 and 21 [19]. These findings suggest that *CCDC69* may be involved in the regulation of adipogenesis. Indeed, the manipulation of *CCDC69* expression via overexpression or knockdown altered the adipogenic differentiation of SGBS cells. Interestingly, Li et al. previously suggested a potential association between *CCDC69* and adipocyte differentiation based on an enrichment analysis of genes strongly correlated with *CCDC69* in breast cancer tissues, which revealed enrichment of genes involved in white adipocyte differentiation, lipid metabolism, and PPARγ signaling [11]. However, the functional role of *CCDC69* in adipogenesis has not been experimentally investigated. Thus, the present findings provide the first experimental evidence that *CCDC69* has a functional role in adipogenic differentiation.

Interestingly, evaluation of adipogenesis on day 9 post-induction revealed that both *CCDC69* overexpression and knockdown resulted in increased lipid accumulation. Analysis of *PPARG* expression showed that *CCDC69* knockdown tended to increase *PPARG* expression from the early stage of differentiation, with a significant increase observed on day 5, suggesting that reduced *CCDC69* expression promotes adipogenic differentiation, at least in part, through enhanced *PPARG* expression. In contrast, *CCDC69* overexpression strongly suppressed *PPARG* mRNA expression during the early phase of adipogenesis and reduced PPARγ protein expression on day 4 after adipogenic induction. These findings suggest that excessive *CCDC69* expression inhibits early adipogenic progression, at least in part, through suppression of *PPARG* expression. However, during the later stages of differentiation, *PPARG* expression in *CCDC69*-overexpressing cells recovered and increased, which was accompanied by enhanced adipogenic differentiation. Collectively, these findings suggest that the effects of CCDC69 on adipogenesis may vary across the differentiation process, although the distinct causal functions of CCDC69 at different stages could not be directly determined in the present study.

During adipogenic differentiation, adipocyte progenitors undergo morphological changes, wherein a rounded morphology or high cell density is associated with enhanced adipogenic commitment and promotes *PPARG* expression and adipogenic differentiation, whereas cell spreading suppresses *PPARG* expression and promotes osteogenic differentiation [23]. Consistent with these observations, *CCDC69*-knockdown SGBS cells exhibited a small and rounded morphology accompanied by increased *PPARG* mRNA expression, whereas cells strongly expressing CCDC69 protein displayed a spread morphology and reduced *PPARG* mRNA expression during the early phase of adipogenesis.

Changes in cell morphology during adipogenesis involve the reorganization of the cytoskeleton [23,24,25,26]. Strong CCDC69 expression caused marked alterations in actin organization, characterized by the disappearance of F-actin stress fibers and the appearance of punctate F-actin structures. These findings suggest that elevated *CCDC69* expression may be associated with a spread cellular morphology. Furthermore, this observation is consistent with previous findings that *CCDC69* regulates microtubule dynamics through KIF2C/MCAK-mediated microtubule depolymerization [9]. The association between CCDC69 expression, cytoskeletal organization, and PPARG expression raises the possibility that cytoskeletal remodeling may be associated with the effects of CCDC69 on adipogenesis. However, the present study did not establish a causal relationship between these events. Conversely, reduced *CCDC69* expression may facilitate the formation of small, rounded cells that are more permissive for adipogenic differentiation.

*CCDC69* expression was almost undetectable before adipogenic induction and gradually increased from day 1, reaching a peak around day 3. This temporal expression pattern may explain the apparent discrepancy between the induction of *CCDC69* during adipogenesis and the enhanced differentiation observed after *CCDC69* knockdown. During the earliest stage of adipogenesis, low *CCDC69* expression may permit cytoskeletal remodeling associated with adipogenic commitment. As adipogenesis progresses, increased *CCDC69* expression may contribute to the later stages of adipocyte maturation through mechanisms distinct from its early effects on cell morphology. Observations in the present study may be consistent with a possible model in which CCDC69 contributes differently to cytoskeletal organization and adipogenic progression at different stages of differentiation. However, the morphological changes observed in this study were examined by overexpressing CCDC69 via adenovirus during the early stage of differentiation when endogenous CCDC69 is barely expressed, and these changes in FLAG-high cells may also reflect supraphysiological effects. An important challenge for future research is to elucidate how dynamic changes in endogenous CCDC69 expression contribute to cytoskeletal reorganization, including the organization of F-actin and tubulin, during adipogenesis.

Although this study demonstrates that CCDC69 regulates adipogenic differentiation and cellular morphology, the established roles of CCDC69 in cytokinesis and microtubule dynamics [8,9] raise the possibility that the observed effects are secondary to changes in cell proliferation or density. However, unlike 3T3-L1 cells, SGBS cells do not necessarily undergo mitotic clonal expansion during adipogenic differentiation [27]. Indeed, neither overexpression nor knockdown of CCDC69 affected cell number during adipogenic differentiation of SGBS cells. These findings indicate that the effects of CCDC69 on adipogenesis are unlikely to be explained simply by changes in cell proliferation or cell density in SGBS cells under the experimental conditions used in this study. Nevertheless, although SGBS cells are a well-characterized model of human preadipocytes, the findings of this study, which were obtained using a single human preadipocyte model, may not fully reflect adipogenesis in normal human adipocytes. Validation in other human preadipocyte models will be important to assess the generalizability of these findings. Furthermore, the molecular mechanisms linking *CCDC69*-mediated cytoskeletal remodeling to PPARG expression remain unclear. Further studies are required to elucidate how changes in microtubule and actin organization induced by *CCDC69* influence adipogenic signaling pathways and adipocyte differentiation.

## 5. Conclusions

Manipulation of CCDC69 expression produces stage-dependent changes in adipogenic markers and is associated with marked alterations in cellular morphology and cytoskeletal organization in SGBS cells. Further studies are needed to determine the generalizability of these findings and to elucidate the molecular mechanisms underlying the relationship between CCDC69-mediated cytoskeletal remodeling and adipogenic signaling.

## Data Availability

The data supporting the findings of this study are contained within the article and its Supplementary Material.

## Supplementary Materials

The following supporting information can be downloaded at: https://www.mdpi.com/article/10.3390/cells15191782/s1.

## Abbreviations

The following abbreviations are used in this manuscript:

CCDC69	coiled-coil domain containing 69
SGBS	Simpson–Golabi–Behmel syndrome
qRT-PCR	quantitative reverse transcription PCR
MOI	multiplicity of infection
shRNA	short hairpin RNA
shCCDC69	short hairpin RNA targeting CCDC69
shNC	negative-control shRNA
DAPI	4,6-diamidino-2-phenylindole
GFP	green fluorescent protein
EDTA	ethylenediaminetetraacetic acid
SDS-PAGE	sodium dodecyl sulfate-polyacrylamide gel electrophoresis
PVDF	polyvinylidene difluoride
PPARγ	peroxisome proliferator-activated receptor gamma
F-actin	filamentous actin
SE	standard error
