# Supplementary material for: CCDC69 Regulates Adipocyte Differentiation and Cellular Morphology in SGBS Cells—A Human Preadipocyte Cell Line
Source: Cells. 2026 Sep 30;15(19):1782. doi: 10.3390/cells15191782 (PMC13664365; doi:10.3390/cells15191782)
Supplement: Supplementary file 1 [file cells-15-01782-s001.zip › Supplementary Figure S1 rev.pdf]

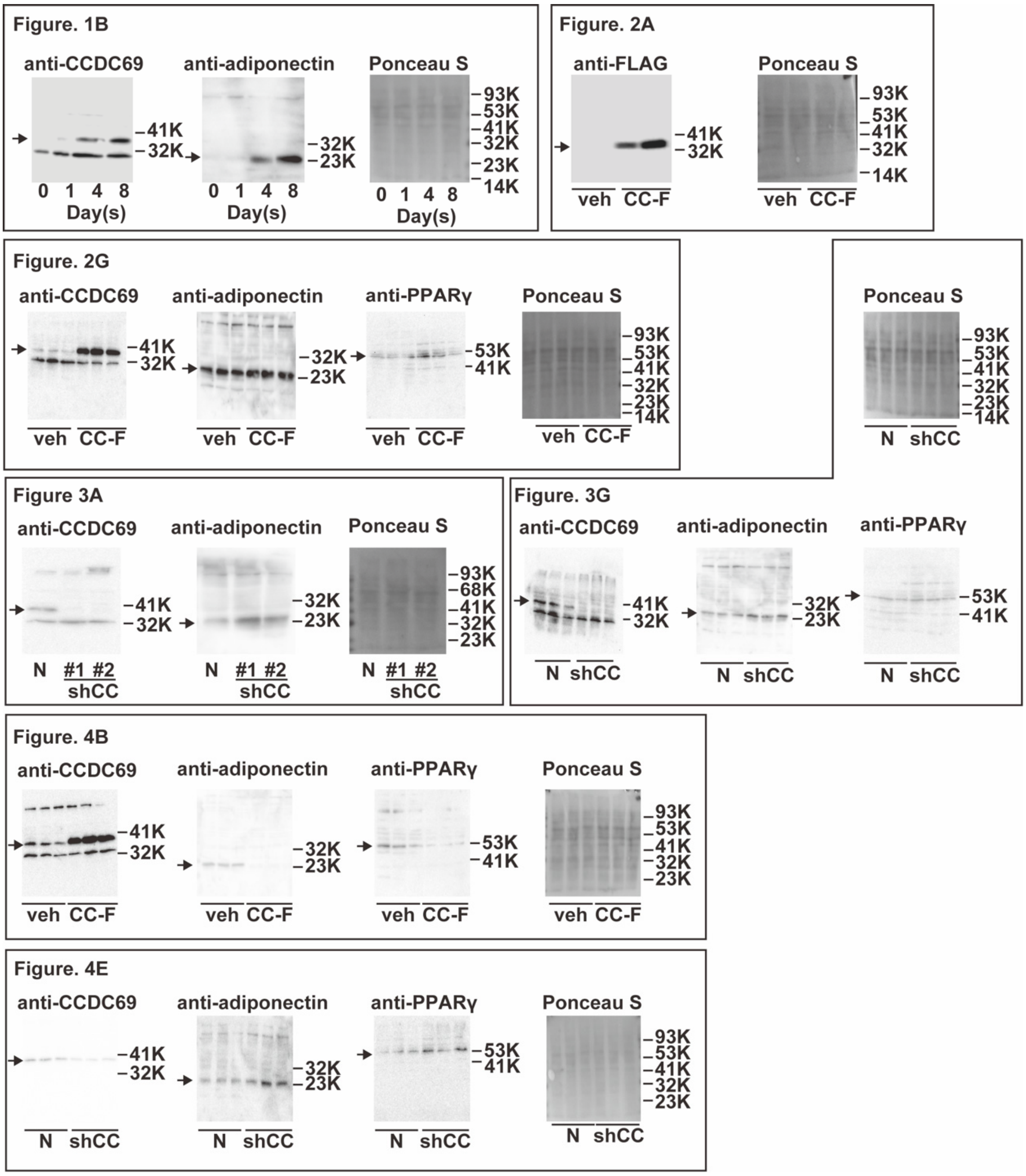

Figure S1. Uncropped Western blot images corresponding to the Western blot data presented in the main figures. Approximate molecular weights are indicated. veh, vehicle; CC-F, CCDC69-FLAG; N, shNC; shCC, shCCDC69. Arrows indicate the bands described in the main text.
