# Supplementary material for: CCDC69 Regulates Adipocyte Differentiation and Cellular Morphology in SGBS Cells—A Human Preadipocyte Cell Line
Source: Cells. 2026 Sep 30;15(19):1782. doi: 10.3390/cells15191782 (PMC13664365; doi:10.3390/cells15191782)
Supplement: Supplementary file 1 [file cells-15-01782-s001.zip › Supplementary Figure S2 rev.pdf]

A

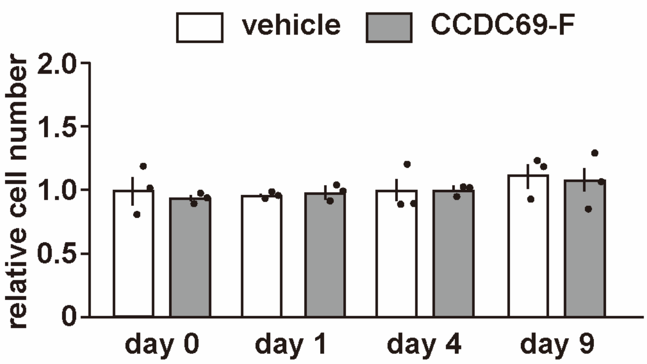

B

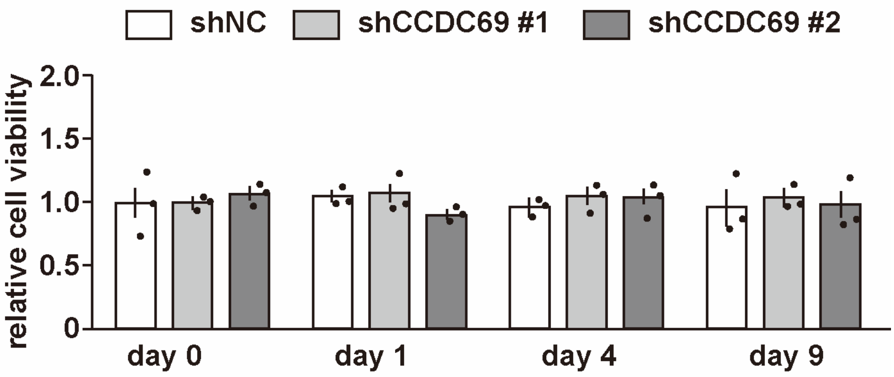

Figure S2. Effects of CCDC69 overexpression (A) or knockdown (B) on cell number during adipogenesis in SGBS cells. SGBS cells were infected with an adenovirus expressing the CCDC69-FLAG fusion protein (CCDC69-F; gray columns in A), shCCDC69 #1 (light gray columns in B), shCCDC69 #2 (strong gray columns in B), or the corresponding control adenovirus (vehicle for A or shNC for B; white columns), followed by adipogenic induction for the indicated days. Values are expressed relative to those in vehicle-treated cells in (A) or shNC-treated cells in (B) on day 0 (set to 1). Individual data points are shown in the graphs. Exact *p* values, effect sizes, 95% confidence intervals, and the number of biological replicates are provided in Supplementary Table S3.
