# Supplementary material for: CCDC69 Regulates Adipocyte Differentiation and Cellular Morphology in SGBS Cells—A Human Preadipocyte Cell Line
Source: Cells. 2026 Sep 30;15(19):1782. doi: 10.3390/cells15191782 (PMC13664365; doi:10.3390/cells15191782)
Supplement: Supplementary file 1 [file cells-15-01782-s001.zip › Supplementary Figure S3 rev.pdf]

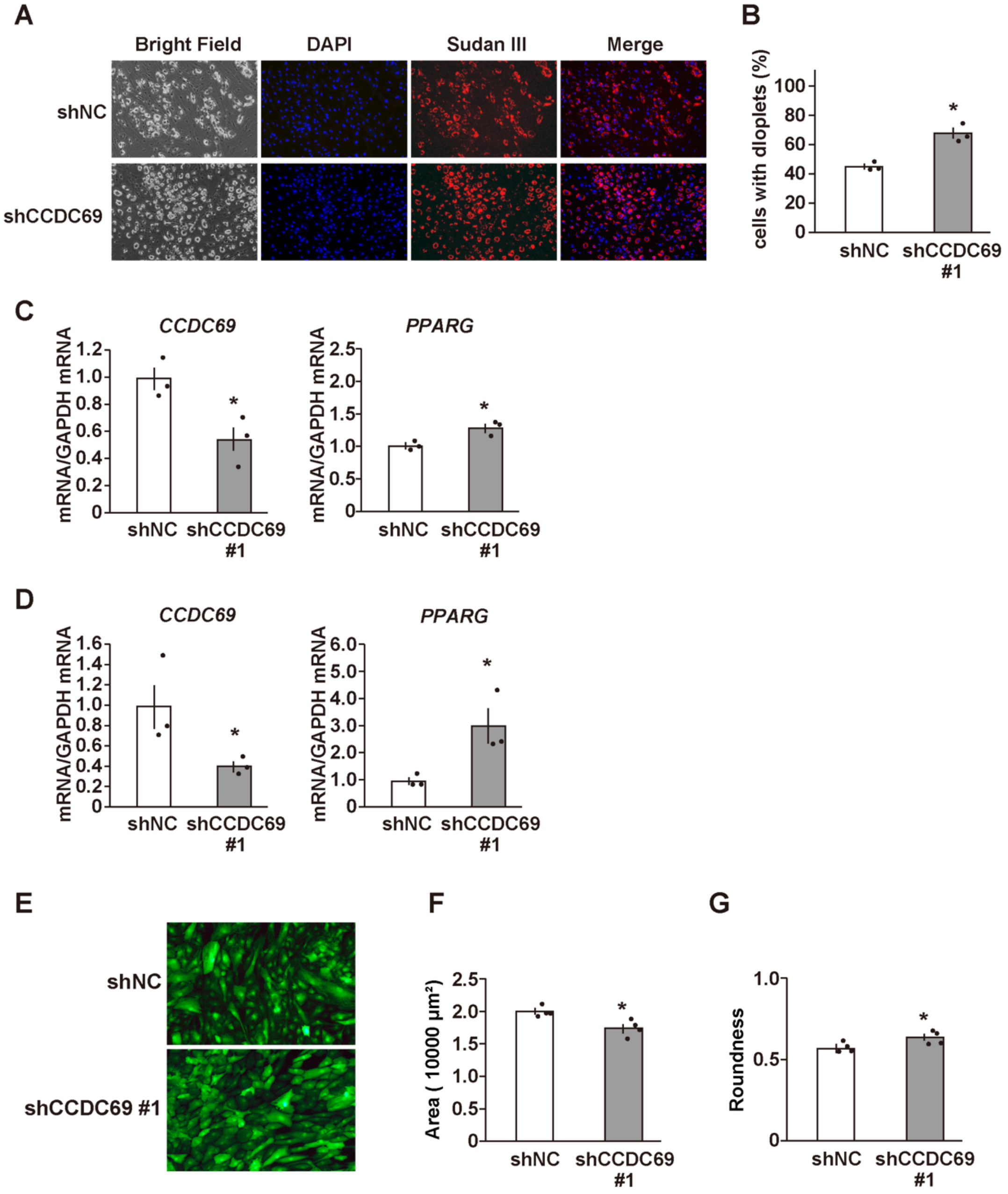

Figure S3. Effects of shCCDC69 #1 expression on adipogenesis in SGBS cells. SGBS cells were infected with adenoviruses expressing shCCDC69 #1 or a negative-control shRNA (shNC) for 24 h, followed by adipogenic induction. (A) Representative images (×100 magnification) of SGBS cells stained with DAPI and Sudan III on day 9 after adipogenic induction. (B) Percentage of cells containing lipid droplets in SGBS cells on day 9 after adipogenic induction. (C, D) Relative mRNA expression of *CCDC69* and *PPARG* in SGBS cells on day 3 (C) and 7 (D) after adipogenic induction. Values are expressed relative to those in shNC-treated cells. (E) Representative images (×100 magnification) of SGBS cells on day 4 after adipogenic induction. (F) Quantification of cell area in SGBS cells expressing shCCDC69 #1 or shNC on day 4 after adipogenic induction. (G) Quantification of cell circularity in SGBS cells expressing shCCDC69 #1 or shNC on day 4 after adipogenic induction. Individual data points are shown in the graphs. Asterisks indicate $p < 0.05$. Exact p values, effect sizes, 95% confidence intervals, and the number of biological replicates are provided in Supplementary Table S3.
