# Supplementary material for: CCDC69 Regulates Adipocyte Differentiation and Cellular Morphology in SGBS Cells—A Human Preadipocyte Cell Line
Source: Cells. 2026 Sep 30;15(19):1782. doi: 10.3390/cells15191782 (PMC13664365; doi:10.3390/cells15191782)
Supplement: Supplementary file 1 [file cells-15-01782-s001.zip › supplementary Table S1.pdf]

Table S1 Nucleotide sequences of primer sets used for qRT-PCR.

Gene	forward primer	reverse primer
human *ADIPOQ*	5'-cctaagggagacatcggtga-3'	5'-gtaaagcgaatgggcatgtt-3'
human *FABP4*	5'-tactgggccaggaatttgac-3'	5'-gtggaagtgacgcctttcat-3'
human *CCDC69*	5'-caagggacagtagccagagc-3'	5'-agacccggatgacagatgag-3'
human *GAPDH*	5'-gaaggtgaaggtcggagtc-3'	5'-gaagatggtgatgggatttc-3'
human *PPARG*	5'-gcaaatgtcaggaccaggtt-3'	5'-tcaaaggagtgggagtggtc-3'
