# Supplementary material for: CCDC69 Regulates Adipocyte Differentiation and Cellular Morphology in SGBS Cells—A Human Preadipocyte Cell Line
Source: Cells. 2026 Sep 30;15(19):1782. doi: 10.3390/cells15191782 (PMC13664365; doi:10.3390/cells15191782)
Supplement: Supplementary file 1 [file cells-15-01782-s001.zip › Supplementary Table S2.pdf]

Table S2 Statistical analysis details for the data presented in the main figures.

Figures		comparison	Statistical test	Effect size	95% CI	*p* value	*n*
Fig.1A	CCDC69		One-way ANOVA	0.98	0.94–1.00	3.62×10^{-7}*	3
	ADIPOQ		One-way ANOVA	0.78	0.37–1.00	5.00×10^{-3}*	3
	CCDC69	day 1 vs day0	Dunnett	6.76	4.42–9.11	4.40×10^{-5}*	3
		day 3 vs day0	Dunnett	15.95	13.60–18.30	3.04×10^{-12}*	3
		day 7 vs day0	Dunnett	10.16	7.81–12.51	5.28×10^{-7}*	3
	ADIPOQ	day 1 vs day0	Dunnett	-9.54×10^{-4}	-2.35–2.35	1.00	3
		day 3 vs day0	Dunnett	0.28	-2.07–2.63	0.97	3
		day 7 vs day0	Dunnett	3.66	1.31–6.00	5.42×10^{-3}*	3
Fig.1C	CCDC69		One-way ANOVA	0.80	0.43–1.00	3.43×10^{-3}*	3
	ADIPOQ		One-way ANOVA	0.93	0.78–1.00	6.66×10^{-5}*	3
	CCDC69	day 1 vs day0	Dunnett	0.28	-2.08–2.63	0.971	3
		day 3 vs day0	Dunnett	4.18	1.78–6.48	2.49×10^{-3}*	3
		day 7 vs day0	Dunnett	2.03	-0.32–4.38	0.09	3
	ADIPOQ	day 1 vs day0	Dunnett	0.48	-1.87–2.82	0.89	3
		day 3 vs day0	Dunnett	3.18	0.83–5.52	0.017*	3
		day 7 vs day0	Dunnett	7.35	5.00–9.69	5.83×10^{-5}*	3
Fig. 2C		vs vehicle	Student's t-test	3.20	0.48–5.81	0.017*	3
Fig. 2E		vs vehicle	Student's t-test	1.80	0.25–3.28	0.021*	5
Fig. 2F	ADIPOQ	vs vehicle	Student's t-test	8.78	2.79–14.85	4.32×10^{-4}*	3
	FABP4	vs vehicle	Student's t-test	5.38	1.45–9.28	2.74×10^{-3}*	3
Fig. 2H	adiponectin	vs vehicle	Student's t-test	2.55	0.15–4.86	0.035*	3
	PPARγ	vs vehicle	Student's t-test	2.91	0.34–5.36	0.024*	3
Fig. 3C	shCCDC69 #1	vs shNC	Student's t-test	4.42	1.58–7.18	0.012*	3
	shCCDC69 #2	vs shNC	Student's t-test	26.8	9.25–43.83	3.24×10^{-4}*	3
Fig. 3E		vs shNC	Student's t-test	9.00	3.86–14.14	6.42×10^{-5}*	4
Fig. 3F	CCDC69	vs shNC	Student's t-test	-27.29	-44.57–-9.41	7.30×10^{-3}*	3
	ADIPOQ	vs shNC	Student's t-test	9.51	3.06–16.04	3.11×10^{-4}*	3
	FABP4	vs shNC	Student's t-test	11.28	3.71–18.96	1.59×10^{-4}*	3
Fig. 3H	adiponectin	vs shNC	Student's t-test	5.55	1.52–9.55	2.44×10^{-3}*	3
	PPARγ	vs shNC	Student's t-test	2.67	0.21–5.00	0.031*	3
Fig.4A	Treatment		Two-way ANOVA	0.32	0.06–1.00	6.56×10^{-3}*	3
	Time		Two-way ANOVA	0.91	0.84–1.00	2.58×10^{-10}*	3
	Treatment×Time		Two-way ANOVA	0.85	0.72–1.00	5.72×10^{-8}*	3
	0 day	vs vehicle	Tukey-HSD	-5.19×10^{-4}	-1.70–1.70	1.00	3
	1 day	vs vehicle	Tukey-HSD	-0.55	-2.26–1.16	0.51	3
	3 day	vs vehicle	Tukey-HSD	-4.08	-6.26–-1.91	6.83×10^{-5}*	3
	5 day	vs vehicle	Tukey-HSD	2.58	0.68–4.49	4.89×10^{-3}*	3
	7 day	vs vehicle	Tukey-HSD	7.59	4.56–10.62	1.06×10^{-8}*	3
Fig. 4C	Adiponectin	vs vehicle	Student's t-test	-15.87	-26.59–-5.37	4.13×10^{-5}*	3
	PPARγ	vs vehicle	Student's t-test	-4.08	-7.20–-0.89	7.48×10^{-3}*	3
Fig.4D	Treatment		Two-way ANOVA	0.69	0.47–1.00	1.69×10^{-6}*	3
	Time		Two-way ANOVA	0.90	0.82–1.00	8.67×10^{-10}*	3
	Treatment×Time		Two-way ANOVA	0.86	0.74–1.00	2.42×10^{-8}*	3
	0 day	vs shNC	Tukey-HSD	-1.20×10^{-3}	-1.70–1.70	1.00	3
	1 day	vs shNC	Tukey-HSD	0.03	-1.68–1.73	0.97	3
	3 day	vs shNC	Tukey-HSD	0.13	-1.57–1.83	0.87	3
	5 day	vs shNC	Tukey-HSD	10.52	6.66–14.39	3.83×10^{-11}*	3
	7 day	vs shNC	Tukey-HSD	1.51	-0.26–3.29	0.079	3
Fig. 4F	Adiponectin	vs shNC	Student's t-test	3.52	0.64–6.32	0.047*	3
	PPARγ	vs shNC	Student's t-test	2.47	0.11–4.71	0.039*	3
Fig. 5E		vs shNC	Student's t-test	-1.79	-3.45–-0.04	0.044*	4
Fig. 5F		vs shNC	Student's t-test	2.64	0.58–4.60	9.71×10^{-3}*	4
Fig. 6B		vs vehicle	Student's t-test	-5.93	-10.17–-1.68	1.19×10^{-3}*	3
Fig. 6D		vs vehicle	Student's t-test	-5.93	-9.85–-1.60	4.42×10^{-3}*	3

Effect size metrics: partial η^2 for ANOVA models; Cohen's *d* for pairwise comparisons. Asterisks indicate $p < 0.05$.
