# Supplementary material for: CCDC69 Regulates Adipocyte Differentiation and Cellular Morphology in SGBS Cells—A Human Preadipocyte Cell Line
Source: Cells. 2026 Sep 30;15(19):1782. doi: 10.3390/cells15191782 (PMC13664365; doi:10.3390/cells15191782)
Supplement: Supplementary file 1 [file cells-15-01782-s001.zip › Supplementary Table S3.pdf]

Table S3 Statistical analysis details for the data presented in the Supplementary figures.

Figures		comparison	Statistical test	Effect size	95% CI	*p* value	*n*
Fig. S2A	Treatment		Two-way ANOVA	1.00×10^{-3}	0.00–1.00	0.69	3
	Time		Two-way ANOVA	0.16	0.00–1.00	0.42	3
	Treatment×Time		Two-way ANOVA	0.02	0.00–1.00	0.94	3
	0 day	vs vehicle	Tukey-HSD	-0.49	-2.23–1.25	0.56	3
	1 day	vs vehicle	Tukey-HSD	0.15	-1.58–1.88	0.86	3
	4 day	vs vehicle	Tukey-HSD	0.028	-1.70–1.76	0.97	3
	9 day	vs vehicle	Tukey-HSD	-0.34	-2.08–1.40	0.68	3
Fig. S2B	Treatment		Two-way ANOVA	0.69	0.47–1.00	0.71	3
	Time		Two-way ANOVA	0.90	0.82–1.00	0.98	3
	Treatment×Time		Two-way ANOVA	0.86	0.74–1.00	0.85	3
	0 day	#1 vs shNC	Tukey-HSD	-0.053	-1.74–1.63	1.00	3
		#2 vs shNC	Tukey-HSD	-0.45	-2.14–1.24	0.84	3
		#1 vs #2	Tukey-HSD	-0.40	-2.09–1.29	0.88	3
	1 day	#1 vs shNC	Tukey-HSD	-0.11	-1.79–1.58	0.40	3
		#2 vs shNC	Tukey-HSD	0.97	-0.74–2.68	0.99	3
		#1 vs #2	Tukey-HSD	1.08	-0.64–2.79	0.47	3
	4 day	#1 vs shNC	Tukey-HSD	-0.49	-2.19–1.20	0.99	3
		#2 vs shNC	Tukey-HSD	-0.38	-2.07–1.31	0.82	3
		#1 vs #2	Tukey-HSD	0.11	-1.58–1.79	0.89	3
	9 day	#1 vs shNC	Tukey-HSD	-0.47	-2.16–1.22	0.84	3
		#2 vs shNC	Tukey-HSD	-1.00×10^{-3}	-1.70–1.68	0.84	3
		#1 vs #2	Tukey-HSD	0.46	-1.23–2.15	1.00	3
Fig. S3B		vs shNC	Student's t-test	4.83	1.22–8.38	4.10×10^{-3}*	3
Fig. S3C	CCDC69	vs shNC	Student's t-test	-2.65	-4.97–-0.20	0.038*	3
	PPARG	vs shNC	Student's t-test	3.10	0.43–5.66	0.019*	3
Fig. S3D	CCDC69	vs shNC	Student's t-test	-1.92	-3.90–-0.20	0.039*	3
	PPARG	vs shNC	Student's t-test	2.39	0.06–4.58	0.043*	3
Fig. S3F		vs shNC	Student's t-test	-2.19	-13.56–-3.68	0.021*	4
Fig. S3G		vs shNC	Student's t-test	2.17	0.29–3.95	0.022*	4

Effect size metrics: partial η^2 for ANOVA models; Cohen's *d* for pairwise comparisons. Asterisks indicate $p < 0.05$.
